# Marine Alkaloids: Compounds with *In Vivo* Activity and Chemical Synthesis

**DOI:** 10.3390/md19070374

**Published:** 2021-06-28

**Authors:** Paulo E. S. Munekata, Mirian Pateiro, Carlos A. Conte-Junior, Rubén Domínguez, Asad Nawaz, Noman Walayat, Elena Movilla Fierro, José M. Lorenzo

**Affiliations:** 1 Centro Tecnológico de la Carne de Galicia, Parque Tecnológico de Galicia, rúa Galicia No. 4, San Cibrao das Viñas, 32900 Ourense, Spain; paulosichetti@ceteca.net (P.E.S.M.); mirianpateiro@ceteca.net (M.P.); rubendominguez@ceteca.net (R.D.); 2 Centro de Tecnologia, Programa de Pós-Graduação em Ciência de Alimentos, Instituto de Química, Universidade Federal do Rio de Janeiro, Avenida Athos da Silveira Ramos 149, Cidade Universitária, Rio de Janeiro 21941-909, RJ, Brazil; carlosconte@id.uff.br; 3 Jiangsu Key Laboratory of Crop Genetics and Physiology, College of Agriculture, Yangzhou University, Yangzhou 225009, China; 007298@yzu.edu.cn; 4 Department of Food Science and Engineering, College of Ocean, Zhejiang University of Technology, Hangzhou 310014, China; Noman.rai66@gmail.com; 5 Complejo Hospitalario Universitario de Ourense, 32005 Ourense, Spain; elena.movilla.fierro@sergas.es; 6 Área de Tecnología de los Alimentos, Facultad de Ciencias de Ourense, Universidad de Vigo, 32004 Ourense, Spain

**Keywords:** animal studies, marine alkaloids, biological activity, cancer, cardiovascular diseases, inflammation, chemical synthesis

## Abstract

Marine alkaloids comprise a class of compounds with several nitrogenated structures that can be explored as potential natural bioactive compounds. The scientific interest in these compounds has been increasing in the last decades, and many studies have been published elucidating their chemical structure and biological effects *in vitro*. Following this trend, the number of *in vivo* studies reporting the health-related properties of marine alkaloids has been increasing and providing more information about the effects in complex organisms. Experiments with animals, especially mice and zebrafish, are revealing the potential health benefits against cancer development, cardiovascular diseases, seizures, Alzheimer’s disease, mental health disorders, inflammatory diseases, osteoporosis, cystic fibrosis, oxidative stress, human parasites, and microbial infections *in vivo*. Although major efforts are still necessary to increase the knowledge, especially about the translation value of the information obtained from *in vivo* experiments to clinical trials, marine alkaloids are promising candidates for further experiments in drug development.

## 1. Introduction

The use of natural products in the treatment and management of human diseases dates back to ancient times. In modern times, the consumption of natural bioactive compounds remains in the habits of modern society as a complementary or alternative strategy to ameliorate the effects of many diseases [1,2]. The role of natural products in human health has been generating increasing interest among researchers, physicians, pharmacists, and professionals of the health sector. This scenario is based on the vast diversity of chemical structures found in natural sources that can be studied as potential drugs or inspire the synthesis of derivatives to improve the management and treatment of diseases at a global scale [3].

Although terrestrial plants are in the focus of numerous studies about bioactive compounds from natural sources, marine organisms have been gaining more space and interest [4]. A growing number of studies have been revealing the rich diversity of compounds found in marine organisms that can be explored as new drugs for human diseases. Among them, alkaloids stand out as one of the most diverse and largely studied groups of compounds [5,6]. This relatively young field of research is growing fast, and many studies have focused on the accurate identification of chemical structures and evaluation of biological potential in vitro. For instance, a recent review compiled around 800 indole alkaloids isolated from algae, microorganisms, sponges, and invertebrate sources [5]. Moreover, several studies have been carried out to screen and characterize the biological activity of marine alkaloids in vitro against cancer [6], inflammatory diseases [7], and parasites [8], for instance.

Although several studies characterized the biological effect of marine alkaloids in vitro, the evaluation at the animal level is a more recent progression to understand the applications and limitations of marine alkaloids. This progression is a step towards the evaluation at the human level that allows a more comprehensive evaluation of the effects that cannot be obtained from chemical methods and cellular models [9]. Thus, this review aims to provide an overview of the current knowledge about the *in vivo* effect of marine alkaloids, the synthetic production of their derivative compounds, and considerations about animal studies with marine alkaloids.

## 2. Marine Alkaloids with Biological Activity *In Vivo*

### 2.1. Antitumor Effect

**Figure 1:**
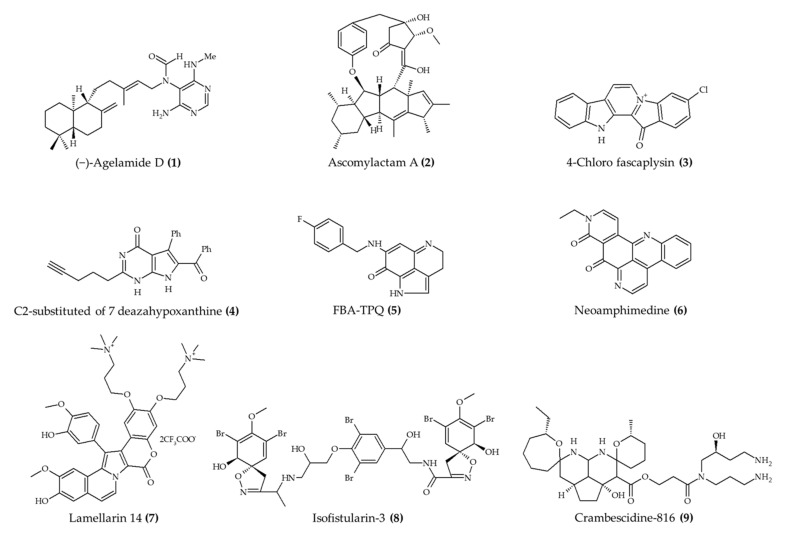
Marine alkaloids with antitumor activity *in vivo*.

The antitumor activity of marine alkaloids has been investigated by many research groups (Table 1). For instance, the recent study carried out by Choi et al. [10] reported the effect of (−)-agelamide D (**1**) (Figure 1) in assisting the treatment of cancer with radiation in mice with xenograft tumors (hepatocellular carcinoma). According to the authors, the treatment with this alkaloid improved the efficiency of radiation therapy wherein the proposed mechanism involved an increase in the stress in the endoplasmic reticulum (increased levels of activating transcription factor 4) and eventual apoptosis during the radiotherapy treatment. In another experiment with mice, Wang et al. [11] explored the effect of ascomylactam A (**2**) against human lung cancer (A549, NCI-H460, and NCI-H1975). The main effects of the treatment with this alkaloid were the significant reduction in the volume and weight of NCI-H460 tumor and the reduction in the volume of A549 tumors in mice treated with 6 mg/kg/day. Conversely, no effect was observed in animals with NCI-H1975 tumors.

A relevant marine alkaloid with antitumor activity is 4-chloro fascaplysin (**3**) [12]. This compound displayed antiangiogenic capacity in mice with human breast cancer MDAMB-231 by reducing the levels of vascular endothelial growth factor (VEGF). Another outcome from this study was the inhibition of Ehrlich ascites carcinoma growth. The authors also observed that 4-chloro fascaplysin (**3**) limited the growth of this carcinoma by activating the phosphoinositide 3-kinases, protein kinase B, and mechanistic target of rapamycin (PI3K/Akt/mTOR) pathway.

A similar inhibitory effect *in vivo* against tumor development was reported by Medellin et al. [13] for C2-substituted 7-deazahypoxanthine (**4**). The animals xenografted with colon cancer SW620 and treated with this alkaloid displayed a significant reduction in total tumor volume. Moreover, no weight loss was observed in the alkaloid-treated group. In the same line of thought, a series of studies against different types of cancer were performed to evaluate the anticancer activity of 7-(4-fluorobenzylamino)-1,3,4,8-tetrahydropyrrolo[4,3,2-de]quinolin-8(1H)-one (FBA-TPQ, compound **5**), which is a synthetic derivative from makaluvamine (naturally found in marine sponge *Zyzzya* sp.) [14]. In the case of breast cancer, this compound reduced tumor growth in athymic nude mice at the three tested levels (5, 10, and 20 mg/kg). However, the authors indicated that animals treated with 10 and 20 mg/kg lost weight during the treatment period. In a subsequent experiment, Chen et al. [15] observed that FBA-TPQ (**5**) slowed the growth of ovarian tumor *in vivo* in a dose-dependent manner (by 20.5% and 69.4% at 1 and 10 mg/kg, respectively). Additionally, no significant changes were observed in the weight of treated animals. According to the authors, this effect was attributed to the formation of radical oxygen species and mitochondrial collapse, which led to apoptosis and interruption of cell cycle progression and proliferation.

Zhang et al. [16] explored the inhibitory effect of FBA-TPQ (**5**) against pancreatic cancer. These authors observed that tumors in animals treated with this alkaloid were reduced gradually during the treatment time and achieved remission at the end of the trial. Moreover, no changes in the weight of animals were observed. The authors also indicated that this effect was attributed to the induction of cell cycle arrest and apoptosis (observed in the in vitro experiments).

The experiment carried out by Marshall et al. [17] is another example of the anticancer effect of marine alkaloids. In this study, these authors observed a significant reduction in the tumor growth (epidermoid-nasopharyngeal and colon cell lines) in mice treated with neoamphimedine (obtained from marine sponge *Xestospongia* sp.; compound **6**). Another relevant synthetic derivative from marine alkaloids with anticancer potential is lamellarin 14 (inspired by naturally occurring lamellarins from mollusk *Lamellaria* sp.; compound **7**) [18]. This compound reduced the growth of adenocarcinoma *in vivo* after 17 days of treatment without significant loss of weight.

Similar to observations in experiments with mouse models, some studies using zebrafish as a model organism indicate the anticancer effect of marine alkaloids. For instance, Florean et al. [19] studied the effect of isofistularin-3 (isolated from marine sponge *Aplysina aerophoba*; compound **8**) against neuroblastoma and prostate cancer. The authors reported that this alkaloid (at 20 and 25 μM) displayed the capacity to inhibit tumor growth by binding to DNA methyltransferase 1 and causing cell cycle arrest (inducing the expression of p21 and p27 cyclin-dependent kinase inhibitors) and apoptosis (favoring the production of tumor necrosis factor). A similar experiment with a zebrafish model indicated that crambescidine-816 (isolated from marine sponge *Crambe crambe*; compound **9**) induced colorectal carcinoma regression [20]. This experiment also revealed that crambescidine-816 (**9**) affected the tumor adhesion capacity, cytoskeletal integrity, and mitochondrial membrane potential leading to apoptosis.

### 2.2. Carodioprotective Activity

**Figure 2:**
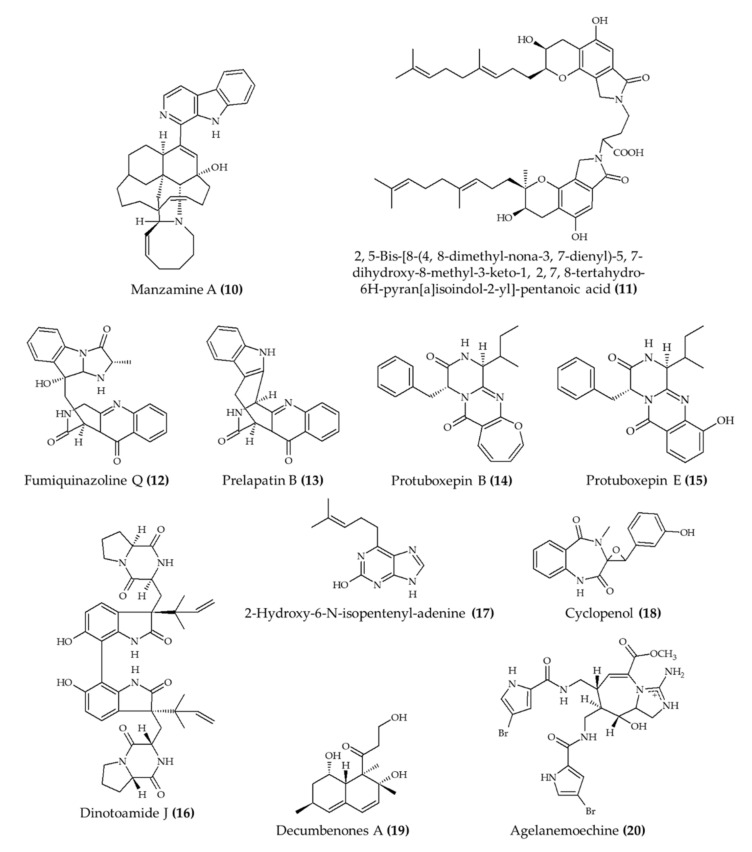
Marine alkaloids with cardioprotective activity *in vivo*.

Marine alkaloids have also been investigated as potential drugs to assist in the management of cardiovascular diseases (Figure 2). In this context, Eguchi et al. [21] explored the effect of manzamine A (naturally found in the marine sponge *Acanthostrongylophora ingens*) in the blood of apoE-deficient mice (naturally prone to developing atherosclerosis). The animals treated with this alkaloid displayed lower blood levels of total, free, and LDL cholesterol and triglyceride than animals in the untreated group. Moreover, manzamine A also reduced the lesions in the arteries of animals in the treated group.

Yan et al. [22] evaluated the thrombolytic effect of fungi fibrinolytic compound 1 (FGC1; 2,5-bis-[8-(4,8-dimethyl-nona-3,7-dienyl)-5,7-dihydroxy-8-methyl-3-keto-1,2,7,8-tetrahydro-6H-pyran[a]isoindol-2-yl]-pentanoic acid; compound **11**). The authors observed a significant improvement in the thrombolysis of pulmonary capillaries and a reduction in the formation of blood clots in mice. Another relevant outcome from this study was the lack of effect in the fibrinogen levels, which indicated that FGC1 (**11**) did not affect the blood coagulation of treated mice. Moreover, the treatment with FGC1 (**11**) also ameliorated the morphological alterations in the walls of the alveolus caused by the induced pulmonary thrombosis.

Interesting outcomes were also obtained in a zebrafish model. Fan et al. [23] explored the effect of several alkaloids obtained from the marine fungus *Penicillium expansum* in controlling blood pressure and inducing angiogenesis. Among all alkaloids, eight compounds displayed the capacity to induce both beneficial effects in the treated group, especially fumiquinazoline Q (**12**), prelapatin B (**13**), and protuboxepin B (**14**) and E (**15**). Likewise, Li et al. [24] observed a significant increase in the formation of new blood vessels in zebrafish embryos treated with alkaloids extracted from marine fungus *Aspergillus austroafricanus*. The alkaloids with the highest angiogenic activity were dinotoamide J (**16**), 2-hydroxy-6-N-isopentenyl-adenine (**17**), cyclopenol (**18**), and decumbenones A (**19**). Similarly, Li et al. [25] observed a similar angiogenic effect in zebrafish treated with agelanemoechine (**20**), especially at 5 μM, from marine sponge *Agelas nemoechinata*.

### 2.3. Antiseizure Activity, Alzheimer’s Disease, and Mental Health

**Figure 3:**
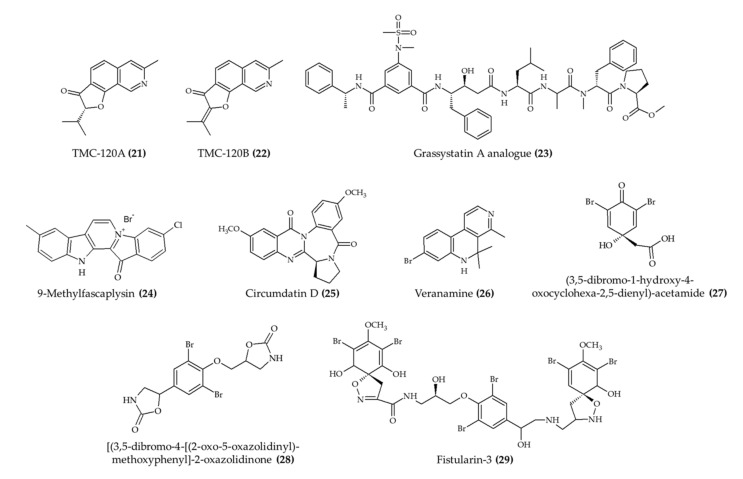
Marine alkaloids with antiseizure activity and health benefits related to Alzheimer’s disease and mental health *in vivo*.

The potential of marine alkaloids to assist in the management of seizures has been explored *in vivo* (Table 2). A recent experiment evaluated the antiseizure effect of TMC-120A and -120B (Figure 3; compounds **21** and **22**, respectively) in both mouse and zebrafish models [26]. In the mouse model, a significant reduction in seizure duration was observed using 10 mg/kg of any of the two alkaloids. Likewise, the duration of seizure behavior and the percentage of animals displaying epileptiform activity in zebrafish model were also reduced by both compounds (20 μg/mL).

Marine alkaloids have also been exerting beneficial effects in the management of Alzheimer’s disease. For instance, the experiment carried out by Liu et al. [27] indicated that a grassystatin A analog (**23**) reduced the levels of amyloid-β peptide (Aβ40) in the brain of treated animals. Likewise, 9-methylfascaplysin (analog of fascaplysin, an alkaloid found in marine sponge *Fascaplysinopsis* sp.; compound **24**) ameliorated the cognitive dysfunction and inhibited the phosphorylation of amyloid-β peptide by tau protein in mice exposed to scopolamine [28]. Additionally, the locomotor function of 9-methylfascaplysin (**24**)-treated animals was not affected. A related experiment with *Caenorhabditis elegans* indicated the role of circumdatin D (**25**) in assisting in the management of Alzheimer’s disease [29]. In this case, temperature-induced paralysis was significantly lower in the group of animals treated with circumdatin D (**25**) in comparison to untreated animals.

Another relevant biological effect related to marine alkaloids is the capacity to exert antidepressant-like activity. This outcome was obtained from the use of veranamine (naturally found in marine sponge *Verongula rigida*; compound **26**) [30]. The main effect was observed in the reduction immobility time of treated animals in relation to untreated ones. However, no significant differences were reported in terms of locomotor activity.

The anxiolytic activity is another interesting effect of marine alkaloids observed *in vivo*. Cesário et al. [31] observed an anxiolytic effect upon exposure to (3,5-dibromo-1-hydroxy-4-oxocyclohexa-2,5-dienyl)-acetamide, [(3,5-dibromo-4-[(2-oxo-5-oxazolidinyl)-methoxyphenyl]-2-oxazolidinone or fistularin-3 (extracted marine sponge *Aplysina fulva*; compounds **27**, **28**, and **29**, respectively) in a zebrafish model. The other six identified alkaloids from the same extract displayed low anxiolytic activity.

### 2.4. Anti-Inflammatory Activity

**Figure 4:**
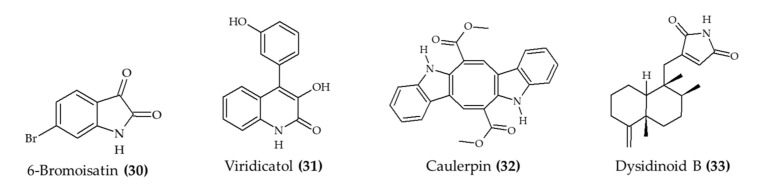
Alkaloids from marine sources associated with anti-inflammatory activity *in vivo*.

The management of the inflammatory process is another relevant health benefit that has been observed for marine alkaloids (Figure 4) *in vivo*. For instance, 6-bromoisatin (obtained from Muricidae mollusk *Dicathais orbita*; compound **30**) exerted a protective effect in the lung tissue of mice with induced inflammation [32]. These results were attributed to the reduction in tumor necrosis factor-α (TNF-α), interleukin-1 beta (IL-1β), and total protein levels in the bronchoalveolar lavage fluid.

The anti-inflammatory effect of marine alkaloids was also observed in the reduction in the allergic reaction induced by ovalbumin [33]. In this case, viridicatol (20 mg/kg; compound **31**) reduced the circulating levels of histamine, mast cell protease-1, and TNF-α, as well as the levels of ovalbumin-specific immunoglobulin E. At the physiological level, the treatment with viridicatol (**31**) also attenuated the lymphocytic infiltration in the jejunum villi and the degranulation of intestinal mast cells. A similar protective effect was reported by Lucena et al. [34], who reported the anticolitis effect of caulerpin (**32**) in mice. These authors observed that a dose of 4 mg/kg ameliorated the damage of the inflammatory response in the colon.

The use of a zebrafish model has also revealed relevant effects of marine alkaloids against inflammation. For instance, dysidinoid B (**33**) reduced the number of inflammatory cells induced by CuSO_4_ at 40 and 80 μM [35]. Similarly, Li et al. [24] reported a significant reduction in the count of macrophages induced by CuSO4 in a mouse model due to the treatment with cyclopenol (**18**), decumbenones A (**19**), or viridicatol (**31**) (extracted from marine fungus *Aspergillus austroafricanus*).

### 2.5. Antiparasitic and Antimicrobial Activity

**Figure 5:**
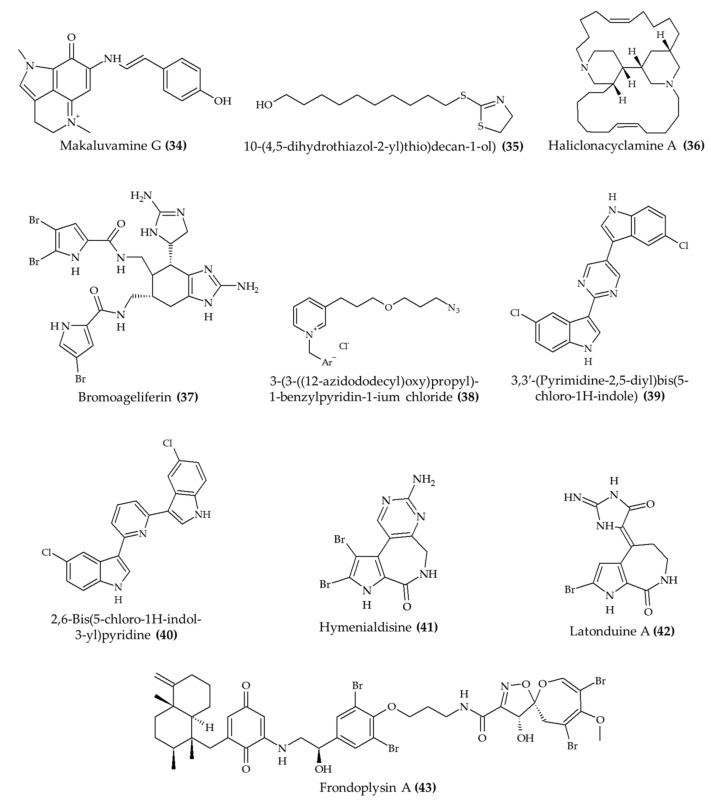
Alkaloids from marine sources with antiparasitic and antimicrobial activity and health benefits related to osteoporosis, cystic fibrosis, and antioxidant activity *in vivo*.

Ameliorating the infection of parasites (Table 3) is another important health benefit associated with marine alkaloids (Figure 5). Davis et al. [36] observed that mice treated with makaluvamine G (obtained from marine sponge *Zyzzya* sp.; compound **34**) had lower blood levels of *Plasmodium berghei* in comparison to animals in the untreated group. Additionally, the authors indicated that the dose administrated to the animals did not induce toxic effects. A similar experiment was carried with 10-(4,5-dihydrothiazol-2-yl)thio)decan-1-ol) (**35**), a derivative of 3-alkylpiridine (naturally found in marine sponge *Theonella* sp.), against the *P. berghei* infection in mice [37]. According to the authors, the treatment with 50 mg/kg suppressed the parasitemia by 20% in comparison to the untreated group. It is noteworthy that this experiment was carried out with an encapsulated material, which is a relevant approach to protect the active compounds during gastric digestion and facilitate the intestinal absorption of compounds administered orally. Likewise, Mani et al. [38] indicated that haliclonacyclamine A (isolated from marine sponge *Haliclona* sp.; compound **36**) reduced *Plasmodium falciparum* level by 45% in mice after 4 days of treatment. Only the treatment with 10 mg/kg increased the survival rate after 18 days of treatment.

Antimicrobial activity is another biologically relevant effect of marine alkaloids. For instance, Pech-Puch et al. [39] evaluated the antimicrobial activity of bromoageliferin (**37**) against *Pseudomonas aeruginosa* in mice. The authors observed a significant increase in the survival rate in the group of animals treated with the marine alkaloid. Similarly, the synthetic 3-(3-((12-azidododecyl)oxy)propyl)-1-benzylpyridin-1-ium chloride (**38**) displayed a protective effect against *Candida albicans* in the kidney and spleen of mice [40]. The marine alkaloids (especially at 1.0 mg/kg) also did not affect the weight of animals during the treatment period. However, no protective effect against *Candida albicans* was observed in the liver.

The antimicrobial activity of marine alkaloids was also reported in the study carried out by Rehberg et al. [41] with methicillin-resistant *Staphylococcus aureus*. These authors observed significant reductions in the size and microbial counts in contaminated skin wounds of animals treated with either 3,3′-(pyrimidine-2,5-diyl)bis(5-chloro-1H-indole) (**39**) or 2,6-bis(5-chloro-1H-indol-3-yl)pyridine (**40**) after 12 days. Additionally, these alkaloids also increased the survival rate of animals intraperitoneally injected with methicillin-resistant *Staphylococcus aureus* by 12 h in relation to control mice (without antibiotic).

### 2.6. Other Health-Related Effects

The biological activity of marine alkaloids *in vivo* is also related to effects other than those aforementioned. Osteoporosis is a disease with a major impact, especially in the elderly population [45]. In this sense, Wang et al. [42] studied the effect of hymenialdisine (Figure 5; compound **41**) in the bone health of mice. The authors observed that treated animals displayed lower levels of bone volume and trabecular thickness levels than nontreated animals. Moreover, this outcome was related to the activation of nuclear factor kappa B (NF-κB); mitogen-activated protein kinase (MAPK); and nuclear factor of activated T-cells, cytoplasmic 1 (NFATc1) signaling pathways.

Marine alkaloids can also assist in the management of cystic fibrosis. This outcome was reported by Carlile et al. [43] for latonduine A (**42**) in a mouse model. These authors observed a significant reduction in the salivary secretion of treated animals. According to the authors, this effect was related to the binding of latonduine A (**42**) to poly(ADP-ribose) polymerases that leads to a correction in the cystic fibrosis transmembrane conductance regulator trafficking to the cell surface. Finally, marine alkaloids can also induce an antioxidant response *in vivo*. Jiao et al. [44] observed a significant increase in the fluorescence of mutant zebrafish treated with frondoplysin A (**43**).

## 3. Chemical Modification and Synthesis of Marine Alkaloids

Several studies described in the last section disclose the potential health benefits of marine alkaloids. Although this scenario is an important advance towards the use of marine alkaloids in disease management, the majority of marine alkaloids do not reach the *in vivo* level. A relevant example that shows the small fraction of compounds with potential biological effects from the huge number of candidates is the screening carried out by Copmans et al. [26]. In this study, more than 2000 marine extracts were screened for *in vivo* characterization of antiseizure activity. Only 43 candidates displayed the target activity and did not induce toxicological effects in both embryonic and larval zebrafish. Eventually, only alkaloids were further evaluated *in vivo*. This study also indicated another important result: 1764 compounds were considered inactive.

This outcome is observed in other natural sources, and major advances are necessary to explore these compounds. Moreover, these compounds with low biological activity may also present low solubility, metabolic instability, and unknown mechanisms of action, and they may be too large for oral administration and gut absorption [46,47]. Consequently, no wide interest from researchers has been generated regarding their pharmacological activity.

One of the possible approaches to overcome this barrier is the modification of the chemical structure of the natural compounds with different substituents and further evaluation of the relationship between each derived compound and the target biological effect [46]. This strategy has been explored in the context of marine alkaloids, and several studies provide valuable information about the structure–activity relationship in vitro [48,49,50,51].

**Figure 6:**
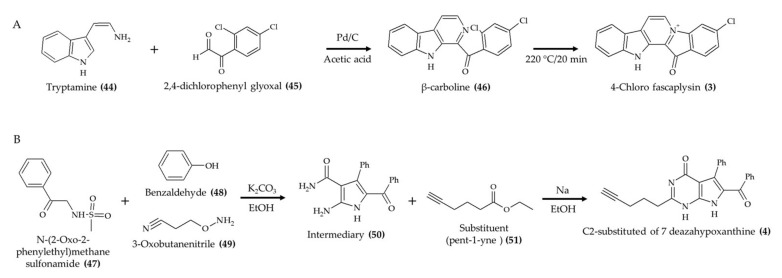
Schematic representation of 4-chlorofascaplysin (**3**) (**A**) and C2-substituted 7-deazahypoxanthine (**4**) (**B**) syntheses (according to Sharma et al. [12] and Medellin et al. [13], respectively).

In terms of derivatives and synthetic compounds inspired from marine alkaloids sources with biological activity *in vivo*, some studies explored interesting modifications. For instance, Sharma et al. [12] evaluated the antitumor effect of the synthetic compound 4-chloro fascaplysin (Figure 6A; compound **3**). The authors synthesized this derivative by reacting tryptamine (**44**) with 2,4-dichlorophenyl glyoxal (**45**) with Pd/C in acetic acid to produce the intermediary compound β-carboline (**46**). Further heating (20 min at 220 °C) led to the generation of 4-chloro fascaplysin (**3**) by intramolecular ring closure. According to the authors, 75% reaction yield was obtained using this route.

Medellin et al. [13] explored the effect of structural modifications in the anticancer activity of rigin D derivatives. The factors evaluated in this study were carbon chain length, elemental composition (Br, N, O, and S), and functional groups (carboxylic acid, phenol, furan, and nitrile) of the substituent group. A schematic representation of the route used by these authors is displayed in Figure 6B. The authors reacted N-(2-Oxo-2-phenylethyl)methanesulfonamide (**47**) with benzaldehyde (**48**) and 3-oxobutanenitrile (**49**) in the presence of potassium carbonate and ethanol under reflux to produce the intermediary compound (**50**). Then, different substituents were coupled to this intermediary compound (**50**) in the presence of sodium in ethanol to produce the rigin D derivatives. According to the authors, increasing chain length reduced the antitumor activity of the rigin D derivatives against HeLa (CCL-2) and MCF-7 cell lines. Conversely, the alkaline substituents with carbon chains containing 5 or 6 C had the most intense inhibitory effects in these cell lines, especially the compound with pent-1-yne substituent (**51**). Additionally, the presence of Br, N, O, S, carboxylic acid, phenol, furan, or nitrile in the carbon chain led to intermediary activity in relation to the large and the 5 or 6 carbon chain groups. Further evaluation *in vivo* confirmed the antitumor potential of the rigin D derivative with pent-1-yne substituent (**4**). Moreover, the authors also indicated that a 72% yield was obtained for this compound.

**Figure 7:**
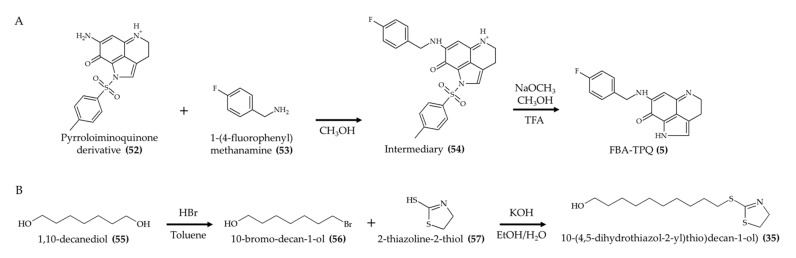
Schematic representation of FBA-TPQ (**5**) (**A**) and 10-((4,5-dihydrothiazol-2-yl)thio)decan-1-ol (**35**) (**B**) syntheses (according to Wang et al. [53] and Guimarães et al. [54], respectively).

FBA-TPQ (**5**) is another bioactive alkaloid obtained by chemical synthesis [16]. In order to produce this compound (Figure 7A), the authors reacted a pyrroloiminoquinone derivative (**52**) [52] with 1-(4-fluorophenyl)methanamine (**53**) in methanol to generate the intermediary compound (**54**). Then, this intermediary compound (**54**) was further reacted with sodium methoxide and methanol in trifluoroacetic acid (TFA) to generate FBA-TPQ (**5**). A previous experiment from this research group indicated a purity of 99% using this route [53]. A related study carried out by Guimarães et al. [54] reported the chemical synthesis 10-((4,5-dihydrothiazol-2-yl)thio)decan-1-ol (Figure 7B; compound **35**). These authors produced this synthetic compound using a two-step reaction. First, 1,10-decanediol (**55**) reacted with HBr in toluene to generate 10-bromo-decan-1-ol (**56**) as an intermediary compound. Then, 10-bromo-decan-1-ol (**56**) was reacted with 2-thiazoline-2-thiol (**57**) and potassium hydroxide in hydroethanolic solution to produce 10-(4,5-dihydrothiazol-2-yl)thio)decan-1-ol) (**35**) with a yield of 97%.

**Figure 8:**
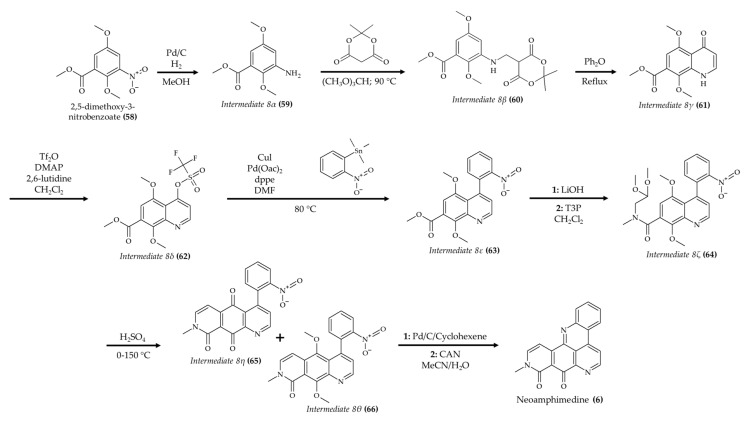
Schematic representation of neoamphimedine (**6**) synthesis (according to Li et al. [55]).

The synthesis of neoamphimedine (**6**) was successfully completed by Li et al. [55]. These authors proposed starting the synthesis with 2,5-dimethoxy-3-nitrobenzoate (**58**) with Pd/C to produce the intermediate 8α (Figure 8; compound **59**). Subsequently, the intermediate 8α (**59**) was treated with Meldrum’s acid and trimethyl orthoformate to generate the intermediate 8β (**60**). Then, the intermediate 8γ (**61**) was produced via thermal ring closure from compound 8β (**60**). In the next step, dry dichloromethane and trifluoromethanesulfonic anhydride with dimethyl amino pyridine (as catalyst) were reacted with compound 8γ (**61**) to generate the intermediate 8δ (**62**). Once this intermediate was obtained, the Stille coupling reaction with trimethyl-(2-nitrophenyl)stannane was carried out using Pd as catalyst to produce the intermediate 8ε (**63**). Then, the intermediate 8ζ (**64**) was produced from a two-step reaction: hydrolyses with lithium hydroxide followed by a coupling reaction with methylamino acetaldehyde dimethylacetal. After this step, an acid-catalyzed ring closure was carried out, which produce the mixture of intermediates 8η and 8θ (quinone (**65**) and dimetoxy (**66**) intermediates, respectively). Finally, the neoamphimedine (**6**) was produced in a two-step reaction: a reduction reaction catalyzed by Pd followed by oxidative demetallation in the presence of ceramic ammonium nitrate. According to the authors, the final step to produce neoamphimedine (**6**) had a yield of 46%. It is noteworthy that this described route comprised fewer steps than another study on the production of neoamphimedine (**6**) published by this research group [56].

**Figure 9:**
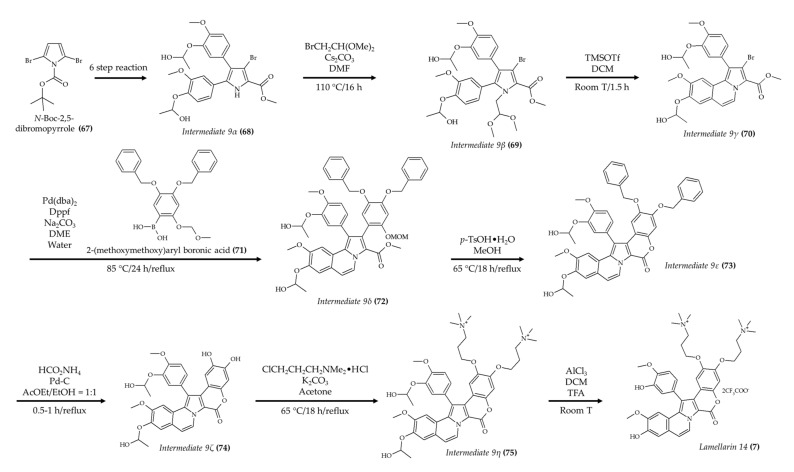
Schematic representation of lamellarin 14 (**7**) synthesis (according to Fukuda et al. [57]).

The synthesis of lamellarin 14 (Figure 9; compound **7**) was developed by Fukuda et al. [57]. These authors initiated the synthesis by carrying out a six-step reaction using *N*-boc-2,5-dibromopyrrole (**67**) to produce the tetra-substituted pyrrole intermediate 9α (**68**) from a previous study [58]. Once this intermediate was obtained, alkylation with bromoacetaldehyde dimethyl acetal at the pyrrole nitrogen was carried out to yield the intermediate 9β (**69**). Then, the tricyclic compound 9γ (**70**) was produced via trifluoromethanesulfonic acid (TfOH)-catalyzed cyclization. This was followed by Suzuki–Miyaura coupling with a 2-(methoxymethoxy)aryl boronic acid (**71**) to produce the intermediate 9δ (**72**). A subsequent direct lactonization of *O*-protected lamellarins was carried out to generate the intermediate 9ε (**73**). Once this intermediate was obtained, hydrogenolysis was performed to remove the *O*-benzyl groups and generate the compound 9ζ (**74**). Then, 3-(dimethylamino)propyl chloride was used to produce the compound 9η (**75**). Finally, the *O*-isopropyl groups were removed to produce lamellarin 14 (**7**). The authors indicated that the last step of lamellarin 14 (**7**) synthesis had a quantitative yield.

**Figure 10:**
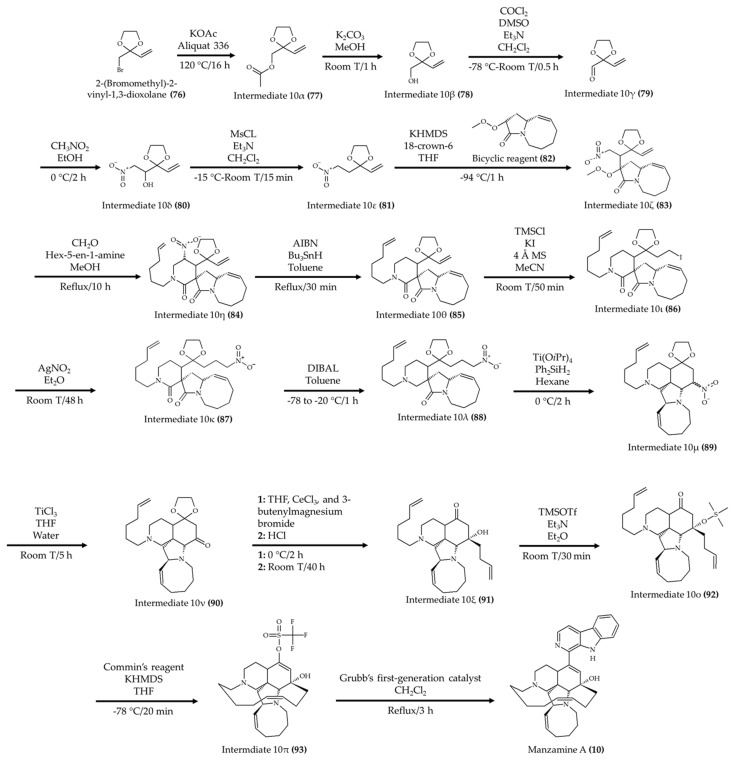
Schematic representation of manzamine A (**10**) synthesis (according to Jakubec et al. [59]).

Manzamine A (**10**) is another marine alkaloid for which chemical synthesis has been achieved. Jakubec et al. [59] synthesized this marine alkaloid by starting with 2-(bromomethyl)-2-vinyl-1,3-dioxolane (**76**) to generate the key intermediate 10ε (Figure 10; compound **81**). Once the compound 10ε (**81**) was obtained, a reaction with a selected bicyclic reagent (**82**) took place to produce intermediate 10ζ (**83**) in the presence of 18-crown-6 and KHMDS. In the following reaction, formaldehyde and hex-5-en-1-amine were used to produce intermediate 10η (**84**). Then, the nitro group was removed from this intermediate to produce the compound 10θ (**85**). Subsequently, the intermediate 10ι (**86**) was produced by adding HI in the double bond via anti-Markovnikov addition. In the next step, silver nitrite was used to produce intermediate 10κ (**87**). Then, a selective reduction with DIBAL and toluene was applied to produce the intermediate 10λ (**88**). After the generation of this intermediate, a new ring was formed by using Ti(OiPr)_4_, Ph_2_SiH_2_, and hexane to produce the compound 10μ (**89**). Subsequently, the intermediate 10ν (**90**) was generated by replacing the NO_2_ group with a ketone. Then, a two-step reaction was carried out to add a 3-butenylcerium-derived organometallic substituent and remove the 1,3-dioxolan-2-ylradical from intermediate 10ν (**90**) and produce compound 10ξ (**91**). In the subsequent step, the intermediate 10ξ (**91**) was reacted with TMSOTf in the presence of Et_3_N and Et_2_O to generate the compound 10ο (**92**). After generating this compound, Commin’s reagent, KHMDS, and THF were added to the reaction to produce the intermediate 10π (**93**), which produced a new ring. Finally, manzamine A (**10**) was formed (52% yield) with Grubb’s first-generation catalyst in the presence of CH_2_Cl_2_ from compound 10π (**93**). The authors also indicated that a yield of 73% was obtained using this route. Moreover, other authors have provided insights for core structures [60] and additional routes for manzamine A (**10**) synthesis [61,62,63].

**Figure 11:**
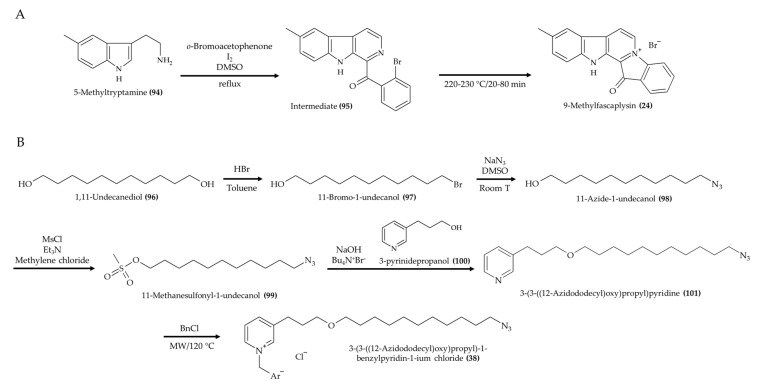
Schematic representation of 9-methylfascaplysin (**24**) (**A**) and 3-(3-((12-azidododecyl)oxy)propyl)-1-benzylpyridin-1-ium chloride (**38**) (**B**) syntheses (according to Pan et al. [28] and Andrade et al. [40], respectively).

The synthesis of 9-methylfascaplysin (Figure 11A; compound **24**) was carried out using 5-methyltryptamine (**94**) with *o*-bromoacetophenone in the presence of I_2_ and DMSO [28]. Once the intermediary adduct product (**95**) was formed, the reaction mixture was heated to obtain 9-methylfascaplysin (**24**) by closing an inner ring. A synthetic route to produce 3-(3-((12-azidododecyl)oxy)propyl)-1-benzylpyridin-1-ium chloride (Figure 11B; compound **38**) was also proposed by Andrade et al. [40]. In this study, the synthesis was initiated with the reaction between 1,11-undecanediol (**96**) and HBr in toluene to yield 11-bromo-1-undecanol (**97**). Then, this compound was reacted with NaN_3_ in DMSO to produce 11-azide-1-undecanol (**98**). Next, the addition of a methanesulfonyl group in this compound was carried out to generate 11-methanesulfonyl-1-undecanol (**99**). Subsequently, 3-(3-((12-azidododecyl)oxy)propyl)pyridine (**101**) was produced by the reaction between 11-methanesulfonyl-1-undecanol (**99**) and 3-pyrinidepropanol (**100**). Finally, the 3-(3-((12-azidododecyl)oxy)propyl)pyridine (**101**) was reacted with BnCl to generate 3-(3-((12-azidododecyl)oxy)propyl)-1-benzylpyridin-1-ium chloride (**38**).

**Figure 12:**
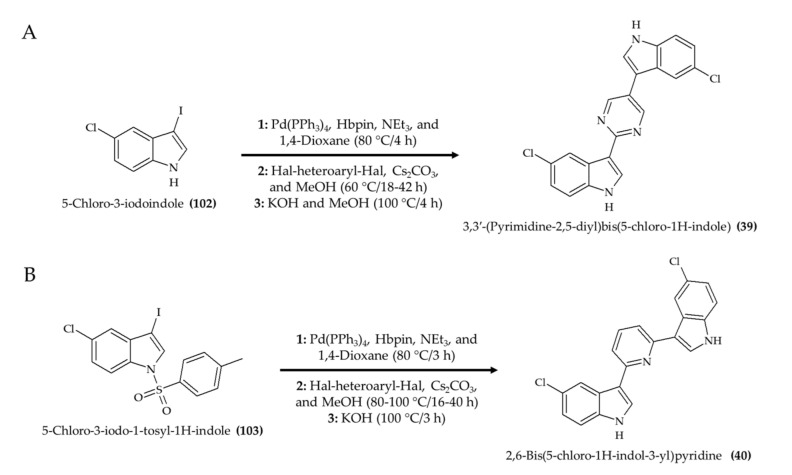
Schematic representation of 3,3′-(pyrimidine-2,5-diyl)bis(5-chloro-1H-indole) (**39**) (**A**)and 2,6-bis(5-chloro-1H-indol-3-yl)pyridine (**40**) (**B**) syntheses (according to Rehberg et al. [41]).

Other relevant synthetic alkaloids are 3,3′-(pyrimidine-2,5-diyl)bis(5-chloro-1H-indole) (**39**) and 2,6-bis(5-chloro-1H-indol-3-yl)pyridine (**40**) [41]. The synthesis of 3,3′-(pyrimidine-2,5-diyl)bis(5-chloro-1H-indole) (Figure 12A; compound **39**) was possible with a one-pot reaction composed of three steps initiated with 5-chloro-3-iodoindole (**102**). A similar strategy was applied in the generation of 2,6-bis(5-chloro-1H-indol-3-yl)pyridine (Figure 12B; compound **40**) from 5-chloro-3-iodo-1-tosyl-1H-indole (**103**).

**Figure 13:**
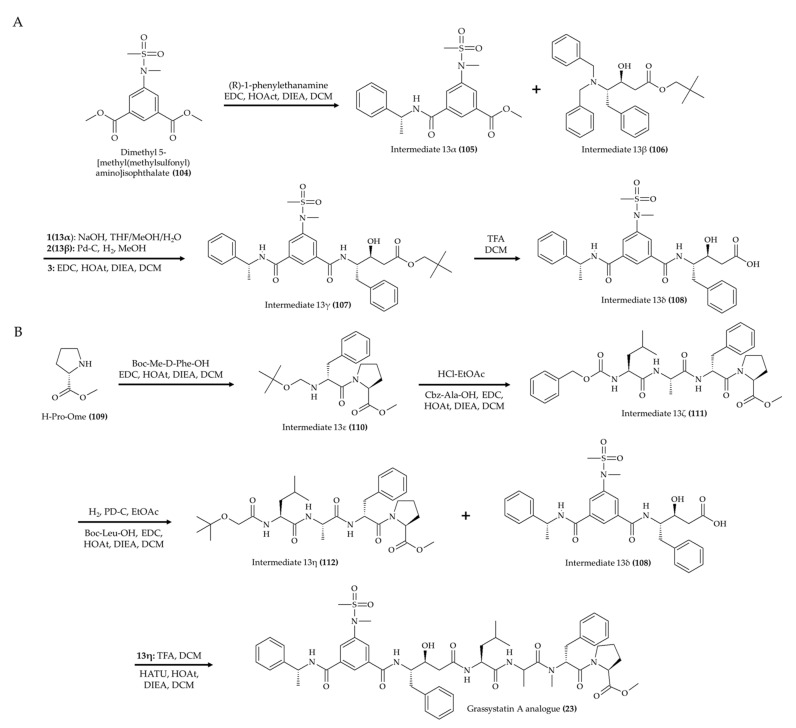
Schematic representation of Grassystatin A analog (**23**) (**A**,**B**) synthesis (according to Liu et al. [27]).

Another interesting synthetic marine alkaloid is the grassystatin A analog (**23**) produced by Liu et al. [27]. These authors proposed a building block strategy to produce this compound by generating the key intermediates 13δ (Figure 13A; compound **108**) and 13η (Figure 13B; compound **112**). In order to obtain the compound 13δ (**108**), the authors initiated the synthesis with dimethyl 5-[methyl(methylsulfonyl)amino]isophthalate (**104**) and carried out a coupling reaction with (R)-1-phenylethanamine to produce the intermediate 13α (**105**). Subsequently, a coupling reaction was performed between intermediate 13α (**105**) and 13β (**106**) with preparatory steps (with NaOH in THF/MeOH/H_2_O and with Pd-C, H_2_, and MeOH for compounds 13α (**105**) and 13β (**106**), respectively). Once these preparatory reactions were accomplished, the coupling reaction was carried out with EDC, HOAt, DIEA, and DCM to generate the compound 13γ (**107**). Then, the key intermediate 13δ (**108**) was produced from compound 13γ (**107**) in TFA and DCM.

The production of key intermediate 13η (**112**) was initiated with the reaction between H-Pro-OMe (**109**) with Boc-Me-D-Phe-OH in the presence of EDC, HOAt, DIEA, and DCM to produce the intermediate 13ε (**110**) [27]. In the next step, this intermediate was treated with HCl-EtOAc and then coupled to Cbz-Ala-OH (in EDC, HOAt, DIEA, and DCM) to generate the compound 13ζ (**111**). Subsequently, the key intermediate 13η (**112**) was generated from the reaction between intermediate 13ζ (**111**) (treated with H_2_, PD-C, and EtOAc) and Boc-Leu-OH (in EDC, HOAt, DIEA, and DCM). Finally, intermediate 13η (**112**) (treated with TFA and DCM) was coupled to intermediate 13δ (**108**) in HATU, HOAt, DIEA, and DCM to produce the grassystatin A analog (reaction yield of 60%; compound **23**).

## 4. Animal Model Context and Further Considerations for Bioactive Marine Alkaloids

The previous sections provide an overview of the current research about the biological effects related to marine alkaloids at the animal level and alternatives to modify their chemical structure. However, it is also important to consider the role of animal models and their application in drug development. Traditionally, animals have been used in the study of diseases and drug development in order to obtain information that cannot be comprehensively determined using in vitro methods (cellular and subcellular/molecular tests), such as the efficacy, potential toxicological effects, and pharmacokinetics [9].

Although different animal models can be considered in the study of diseases, such as mice [64], rabbits [65], pigs [66], and fish [67], selecting an appropriate model to study is a complex and multifactorial challenge. Ideally, an animal model should provide a replication of the same elicitors, symptoms, physiological changes, therapeutic responses, resolution mechanisms, and side effects observed in humans. Since it is not possible to carry out an experiment and comply with all these conditions, understanding the similarities and limitations of each animal model is a necessary step to strengthen the progression of in vitro data to more complex organisms and the conclusions from these studies [9].

In this line of thought, Denayer et al. [68] discuss these aspects and advise considering multiple aspects for validating an animal model for drug development: species similarity (human > nonhuman primate > nonhuman mammal > nonmammal), complexity of the model (*in vivo* > tissue > cellular > subcellular/molecular), disease simulation (true > complex > pharmacological > no), face validity (more than one core symptom > one core symptom > one symptom > no), and predictivity (graded for all pharmacology principles > graded for certain pharmacology principles > all or none for certain pharmacology principles > no or not shown).

Another important aspect in animal trials is the humanization of animal models in order to increase the similarities of specific conditions to human physiological characteristics, pathologies, and metabolic processes. The development of mutant strains of animals with specific genetic modifications to match with these necessities in animal studies is the most common approach [69]. Additionally, some specific cases, such as the tumor xenograft in cancer studies, can be cited [70].

To the best of our knowledge, only two animal models have been applied to test marine alkaloids: mice and zebrafish (Table 1, Table 2 and Table 3). In the case of mice, this animal model has been widely applied for several diseases. Mice are the most widely used animals for disease studies and drug development. Some of the advantages associated with choice are the ease of breeding, availability of many strains, genetic modification, relatively low cost of maintenance, and small quantity of tested compounds [68,71]. Moreover, mice have been used as an animal model for many diseases, such as cancer [72], cardiovascular diseases [73], and seizures [74].

In a similar way, the zebrafish (*Danio rerio*, a teleost species or bony fish) has emerged as a potential animal model in the studies of diseases and drug development. This animal model has relevant molecular and physiological processes that are similar to those observed in humans and can be explored to discover and characterize the biological properties of new drugs such as marine alkaloids. In general, the embryonic, larval, and adult phases of zebrafish have been used in drug discovery. Interesting aspects related to the zebrafish model are its high fecundity, fast growth, relatively low maintenance cost, small size (either as embryos or adults), fluorescent receptor production, and optical clarity (allowing the visualization of internal organs and structures) [75,76,77]. Zebrafish have been applied in the context of studying specific diseases such as cancer [78,79,80], cardiovascular diseases [81], inflammatory diseases [75,76], and neurological disorders and mental health [82].

However, animal models may not provide the whole spectrum of physiological changes associated with a disease [9]. Moreover, each model has limitations that limit or preclude its use. In the case of mice, the limitations are known to be disease- and process-specific due to the long history and widespread utilization of these animals in disease and drug development studies [83,84]. For the zebrafish model, the limitations are mainly related to the respiratory, reproductive, and locomotion systems; small organ size; and the aquatic habitat [85].

An interesting approach to overcome the individual limitations of each animal model is the evaluation using more than one animal model in order to obtain a more comprehensive, detailed, and better replication of a given human disease [68]. For instance, Copmans et al. [26] explored the use of both mice and zebrafish models to test the antiseizure effect of TMC-120A (**21**) and TMC-120B (**22**). The results from both animal models indicate a congruent interpretation of the potential biological effect to manage seizures. Therefore, it is necessary to increase efforts to explore different animal models, whenever possible, by means of combining strengths and reducing the limitations of each animal model in the area of marine alkaloid drug development.

Finally, it is worth commenting that the translation of results and conclusions from animal studies and successful clinical application is another factor that imposes a persistent necessity to improve animal studies. The rate of translation of animal study results to clinical experiments is low, and such studies may not effectively predict the biological effect in humans. Different authors have indicated that less than 10% of all experiments are translated into actual drugs [9,86,87]. In addition, failure to provide the target therapeutic effect in patients at clinical levels is also accompanied by the concern of generation of acute symptoms and side effects not observed at the animal level [9]. The marine alkaloids included in this review can be considered as potential candidates to manage and treat some of the main diseases imposing a heavy burden on our society. Proper and solid information must be generated in order to reduce misinterpretation and the raising of premature expectations in further clinical evaluation.

## 5. Conclusions

The progression of scientific knowledge about the biological effects of marine alkaloids and their derivatives (especially those obtained from chemical synthesis) in vitro has been also observed *in vivo*. An increasing number of studies indicate that these compounds can exert beneficial effects in complex organisms such as mice and zebrafish. Although each animal model has its own limitations, both are valuable models to obtain information and improve the knowledge about the protective effects of the mechanisms activated by marine alkaloids and should be seen as complementary models rather than competitive. However, major efforts are still necessary to improve the validation and define the actual role of the zebrafish model in animal drug discovery.

Further experiments *in vivo* should target other relevant diseases such as stroke, respiratory diseases, diabetes, and Alzheimer’s disease. The studies in chemical synthesis are also relevant for marine alkaloids in order to obtain active compounds from relatively inactive ones. Additionally, the synthetic production of those compounds with promising effects for clinical trials is also advised in order to circumvent the exploration of natural sources and preserve the marine environment.

## Figures and Tables

**Table 1:** Antitumor and cardiovascular protective activities of marine alkaloids and derivative compounds evaluated *in vivo*.

Compound and Source	Experimental Conditions	Effect	Ref.
Anticancer activity			
(−)-Agelamide D (**1**) from marine sponge *Agelas* sp.	6–7-week-old male Balb/c nude mice; intraperitoneal injection; 1.25 mg/kg/day; 3 times a week; mice euthanized after 21 days	Increased the efficacy of radiation therapy in xenograft Hep3B cells with reduced systemic toxicity	[10]
Ascomylactam A (**2**) from mangrove endophytic fungus *Ascomycota* sp.	Male BALB/c-nu mice; intraperitoneal injection; 3 and 6 (A549 and NCI-H460), 5 or 10 (NCI-H1975) mg/kg/day; every 3 days for 21 days	Suppression of A549 (6 mg/kg/day; volume) and NCI-H460 (6 mg/kg/day; volume and weight) and lung tumor growth; no effect in NCI-H1975 tumor	[11]
Synthetic 4-chloro fascaplysin (**3**) (found in marine sponge)	4–6-week-old C57B/6J female mice; oral administration; 1, 3, and 5 mg/kg/day for 5 days (tumor angiogenesis assay); 4 and 7 mg/kg/day for 8 days (Ehrlich solid tumor model)	Reduced the formation of VEGF-mediated microvessels and blood vessel formation of xenograft breast cancer cells (1, 3, and 5 mg/kg/day) and Ehrlich solid tumor formation (4 and 7 mg/kg/day)	[12]
Synthetic derivative (C2-substituted) of 7-deazahypoxanthine (**4**) from rigidin D	4–6-week-old female athymic nude mice; intraperitoneal injection; 3 mg/kg; 5 times per week for 17 days	Reduced tumor growth; no effect on weight	[13]
Synthetic FBA-TPQ (**5**); makaluvamine analog from marine sponge *Zyzzya* sp.	4–6-week-old female athymic nude mice; intraperitoneal injection; 5 mg/kg/day, 3 days/week for 3 weeks; 10 mg/kg, 3 days/week for 2 weeks; and 20 mg/kg, 3 days/week for 1 week	Reduced tumor growth; weight loss with doses of 10 and 20 mg/kg	[14]
Synthetic FBA-TPQ (**5**); makaluvamine analog from marine sponge *Zyzzya* sp.	5-week-old female athymic nude mice; intraperitoneal injection; 1 and 10 mg/kg/day; 5 days/week for two and half weeks	Reduced tumor growth and no significant effect on body weight	[15]
Synthetic FBA-TPQ (**5**); makaluvamine analog from marine sponge *Zyzzya* sp.	4–6-week-old female athymic nude mice; intraperitoneal injection; 5 and 10 mg/kg/day; 5 days per week for 3 weeks	Reduced tumor growth and induced remission	[16]
Neoamphimedine (**6**) from marine sponge *Xestospongia* sp.	Nude mice; intraperitoneal injection; 50 mg/kg; 4 q.d.	Reduced tumor growth	[17]
Synthetic lamellarin 14 (**7**); lamellarin analog from mollusk *Lamellaria* sp	BALB/c nu/nu mice; 5 and 10 mg/kg; once a day for 17 days	Reduced tumor growth with no effect on body weight	[18]
Isofistularin-3 (**8**) from marine sponge *Aplysina aerophoba*	Zebrafish embryos; 15, 20, and 25 μM; incubation for 24 h	Reduced neuroblastoma (15–25 μM) and prostate (20 and 25 μM) cancer development	[19]
Crambescidine-816 (**9**) from marine sponge *Crambe crambe*	Zebrafish embryos; 0.5, 1, and 2 μM; 48 h after fertilization	Reduced tumor development; no effect on survival rate	[20]
Cardioprotective activity			
Manzamine A (**10**) from marine sponge *Acanthostrongylophora ingens*	6-week-old apoE-deficient mice; oral administration; 30 mg/kg for 80 days	Reduced total, free, and LDL cholesterol and triglyceride levels; atherosclerotic lesions were diminished	[21]
Fungi fibrinolytic compound 1 (**11**) from marine fungus *Stachbotrys longispora* FG216	Wistar rats; 5, 10, and 25 mg/kg (morphological effect in lungs; euglobulin lysis time); injection in caudal vein	Reduced morphological changes from induced thrombosis and reduced euglobulin lysis time; no effect on fibrinogen degradation concentrations (5–25 mg/kg)	[22]
4 alkaloids (**12–15**) from marine fungus *Penicillium expansum*	Zebrafish embryos; 1, 10, and 100 μg/mL; incubation for 24 h	Reduced bradycardia; induced angiogenesis	[23]
4 alkaloids (**16–19**) from marine fungus *Aspergillus austroafricanus*	Zebrafish embryos; 30, 70, 120 µg/mL; incubation for 24 h	Induced angiogenesis	[24]
Agelanemoechine (**20**) from marine sponge *Agelas nemoechinata*	Zebrafish embryos; 1.25, 2.5, 5, 10, and 20 μM	Induced angiogenesis	[25]

n.i.: not indicated; FBA-TPQ (**5**): 7-(4-fluorobenzylamino)-1,3,4,8-tetrahydropyrrolo[4,3,2-de]quinolin-8(1H)-one.

**Table 2:** Effects of marine alkaloids and derivative compounds on seizures, Alzheimer’s disease, mental health, and inflammation evaluated *in vivo*.

Compound and Source	Experimental Conditions	Effect	Ref.
Antiseizure activity, Alzheimer’s disease, and mental health			
TMC-120A (**21**) and TMC-120B (**22**) isoquinoline alkaloids from marine fungus *Aspergillus insuetus*	Male NMRI mice; intraperitoneal injection; TMC-120A (1.25, 2.5, 5, and 10 mg/kg) and TMC-120B (2.5, 5, 10, and 20 mg/kg); 30 min before electrical stimulation	Reduced seizure duration (at 10 mg/kg for both alkaloids)	[26]
TMC-120A (**21**) and TMC-120B (**22**) isoquinoline alkaloids from marine fungus *Aspergillus insuetus*	Zebrafish larvae; 5, 10, and 20 μg/mL; 2 h incubation	Reduced the proportion of animals and seizure duration (20 μg/mL)	[26]
1 alkaloid (**23**); grassystatin A analog from marine cyanobacterium *Symploca* sp.	8-week-old CF-1 mice; intraperitoneal injection; 30 mg/kg; single day	Reduced the Aβ40 level in the brain	[27]
9-Methylfascaplysin (**24**); Fascaplysin analog from marine sponge *Fascaplysinopsis* sp.	4-month-old male ICR mice; intrahippocampal injection; scopolamine with 7.3 ng and 21.9 ng; once a day for 10 days	Ameliorated cognitive dysfunction; inhibited Aβ-induced tau hyperphosphorylation; no effect in locomotor function	[28]
Circumdatin D (**25**) from marine fungus *Aspergillus ochraceus*	*Caenorhabditis elegans*; 50, 100, and 200 mM incubation for 36 h	Reduced paralysis rate	[29]
Veranamine (**26**) from marine sponge *Verongula rigida*	Male Swiss Webster mice; intraperitoneal injection; 20 mg/kg; single day	Reduced immobility time; no effect on locomotor activity	[30]
3 alkaloids (**27–29**) from marine sponge *Aplysina fulva*	Zebrafish adult; 0.1, 0.5, and 1.0 mg/mL; 1 h incubation	Induced anxiolytic effect and involved the GABAergic system	[31]
Anti-inflammatory			
6-Bromoisatin (**30**) isolated from Muricidae mollusk *Dicathais orbita*	Male and female C57 black/6 mice; oral administration; 0.5 and 0.1 mg/g HBG extract and 0.05 and 0.1 mg/g 6-bromoisatin (**30**); 48, 24, and 1 h prior to the administration of LPS; mice euthanized after 3 h	Reduce acute lung inflammation, TNFα, IL-1β, and total protein levels in BALF, attenuated physiological changes	[32]
Viridicatol (**31**) isolated from marine fungus *Penicillium griseofulvum*	6–7-week-old female BALB/c mice; oral administration; 5, 10, and 20 mg/kg for 13 days	Reduced OVA-specific IgE, serum histamine, mMCP-1, and TNF-α; increased IL10 level	[33]
Caulerpin (**32**) from seaweed *Caulerpa racemosa*	6–8-week-old male C57BL/6 mice; oral administration; 0.4, 4, and 40 mg/kg for 7 days	Reduced colon damage and shortening and DAI (4 mg/kg)	[34]
Dysidinoid B (**33**) from marine sponge *Dysidea septosa*	Zebrafish embryos; 20, 40, and 80 μM; incubation for 2 h	Reduced inflammation (40 and 80 μM)	[35]
3 alkaloids (**18**, **19,** and **31**) from marine fungus *Aspergillus austroafricanus*	Zebrafish embryos; 30, 70, and 120 µg/mL; incubation for 2 h	Induced anti-inflammatory response	[24]

Aβ: amyloid-β peptide; BALF: bronchoalveolar lavage fluid; DAI: disease activity index; HBG: hypobranchial gland; IL10: interleukin 10; IL-1β: interleukin-1 beta; LPS: lipopolysaccharide; mMCP-1: mast cell protease-1; OVA: ovalbumin; TNF-α: tumor necrosis factor-α.

**Table 3:** Antiparasitic and antimicrobial activity and health benefits related to osteoporosis, cystic fibrosis, and antioxidant activity of marine alkaloids *in vivo*.

Compound and Source	Experimental Conditions	Effect	Ref.
Antiparasitic activity			
Makaluvamine G (**34**) from marine sponge *Zyzzya* sp.	7–9-week-old male and female Swiss albino mice; subcutaneous injection; 8 mg/kg/day for 4 days	Reduced the growth of *Plasmodium berghei* in infected mice	[36]
10-(4,5-Dihydrothiazol-2-yl)thio)decan-1-ol) (thiazoline) (**35**), a synthetic analog of 3-alkylpiridine	6–8-week-old female C57BL/6 mice; oral administration; 25 and 50 mg/kg (nanoemulsion) and 25 mg/kg (free compound); 4 h after infection for 4 days; samples collected after 5, 8, and 10 days	Reduced parasitemia for 8 days (especially with 50 mg/kg)	[37]
Haliclonacyclamine A (**36**) from marine sponge *Haliclona* sp.	Swiss female mice; 0.1, 1, and 10 mg/kg; once a day for 4 days	Reduced parasitemia by 45% after 4 days of treatment (10 mg/kg)	[38]
Antimicrobial activity			
Bromoageliferin (**37**) from marine sponge *Agelas dilatata*	*Galleria mellonella* larvae; 2 mg/kg	Increased survival rate after infection with *Pseudomonas aeruginosa*	[39]
3-(3-((12-Azidododecyl)oxy)propyl)-1-benzylpyridin-1-ium chloride (**38**), a synthetic 3-alkylpiridine analog	6–8-week-old Swiss male mice; 0.5 and 1.0 mg/kg; 2, 24, and 48 h postinfection	Reduced microbial infection in kidney and spleen (1.0 mg/kg); no effect in liver	[40]
2 alkaloids (**39** and **40**), hyrtinadine A analogs from Red Sea sponge *Hyrtios* sp.	BALB/c female mice; topical administration and intraperitoneal injection; 5 mg/kg; single application	Improved wound skin wound healing; increased survival rate	[41]
Osteoporosis			
Hymenialdisine (**41**) from marine sponge	11-week-old female C57BL/6j mice; intraperitoneal injection; 1 mg/kg; every 2 days for 6 weeks	Reduced the loss of bone volume and trabecular thickness	[42]
Cystic fibrosis			
Latonduine A (**42**) from marine sponge *Stylissa carteri*	10–12-week-old F508del-CFTR homozygous mice; gavage; 50 mg/kg once daily for 2 days	Reduced salivary secretion	[43]
Antioxidant activity			
Frondoplysin A (**43**) from marine sponge *Dysidea frondosa*	Zebrafish embryos; 20 μM	Induced antioxidant response	[44]

## Data Availability

Data sharing is not applicable to this article.
